# A Text Messaging Intervention for Priming the Affective Rewards of Exercise in Adults: Protocol for a Microrandomized Trial

**DOI:** 10.2196/46560

**Published:** 2023-09-01

**Authors:** Sonali R Mishra, Walter Dempsey, Predrag Klasnja

**Affiliations:** 1 Department of Internal Medicine University of Michigan Ann Arbor, MI United States; 2 Department of Biostatistics University of Michigan Ann Arbor, MI United States; 3 School of Information University of Michigan Ann Arbor, MI United States

**Keywords:** mobile health, mHealth interventions, physical activity, affective attitudes, mobile phone

## Abstract

**Background:**

Physical activity is a critical target for health interventions, but effective interventions remain elusive. A growing body of work suggests that interventions targeting affective attitudes toward physical activity may be more effective for sustaining activity long term than those that rely on cognitive constructs alone, such as goal setting and self-monitoring. Anticipated affective response in particular is a promising target for intervention.

**Objective:**

We will evaluate the efficacy of an SMS text messaging intervention that manipulates anticipated affective response to exercise to promote physical activity. We hypothesize that reminding users of a positive postexercise affective state before their planned exercise sessions will increase their calories burned during this exercise session. We will deploy 2 forms of affective SMS text messages to explore the design space: low-reflection messages written by participants for themselves and high-reflection prompts that require users to reflect and respond. We will also explore the effect of the intervention on affective attitudes toward exercise.

**Methods:**

A total of 120 individuals will be enrolled in a 9-week microrandomized trial testing affective messages that remind users about feeling good after exercise (40% probability), control reminders (30% probability), or no message (30% probability). Two types of affective SMS text messages will be deployed: one requiring a response and the other in a read-only format. Participants will write the read-only messages themselves to ensure that the messages accurately reflect the participants’ anticipated postexercise affective state. Affective attitudes toward exercise and intrinsic motivation for exercise will be measured at the beginning and end of the study. The weighted and centered least squares method will be used to analyze the effect of delivering the intervention versus not on calories burned over 4 hours around the time of the planned activity, measured by the Apple Watch. Secondary analyses will include the effect of the intervention on step count and active minutes, as well as an investigation of the effects of the intervention on affective attitudes toward exercise and intrinsic motivation for exercise. Participants will be interviewed to gain qualitative insights into intervention impact and acceptability.

**Results:**

Enrollment began in May 2023, with 57 participants enrolled at the end of July 2023. We anticipate enrolling 120 participants.

**Conclusions:**

This study will provide early evidence about the effect of a repeated manipulation of anticipated affective response to exercise. The use of 2 different types of messages will yield insight into optimal design strategies for improving affective attitudes toward exercise.

**Trial Registration:**

ClinicalTrials.gov NCT05582369; https://classic.clinicaltrials.gov/ct2/show/NCT05582369

**International Registered Report Identifier (IRRID):**

PRR1-10.2196/46560

## Introduction

### Background

Exercising is crucial for maintaining and improving health and fitness and is linked to improvements in both physical and mental health [1,2]. However, only 53% of Americans [3] meet the recommended threshold of 150 active minutes a week [4], and only 23% meet guidelines for both muscle strengthening and aerobic exercise [3], creating a critical need for effective interventions to help Americans exercise. Health researchers have explored ways to leverage technologies such as smartphones and wearables to meet this need, devising interventions that use displays, messages, and other technological channels to encourage physical activity [5]. Reviews of this extensive body of work have found that mobile interventions hold promise [6,7], with a correlation between engagement with the mobile health (mHealth) intervention and efficacy [7]. However, the effects of mHealth interventions on physical activity thus far have often been relatively modest [5,8], when they are effective at all [9,10]. In addition, effects generally do not last long term [11].

Most existing mHealth interventions use a limited set of strategies to encourage physical activity [12,13]. In particular, many interventions rely on goals- and self-efficacy–related strategies, such as supporting goal setting and self-monitoring, providing feedback on performance [5,8], offering rewards [14], and providing some form of social support [8]. However, this range of approaches is relatively narrow compared with a full taxonomy of behavior change techniques [15]. One review found that only 39 out of 93 techniques were used across all top physical activity mobile apps [12]. One potential avenue for improving the efficacy of physical activity mHealth interventions is to devise interventions that expand the range of the targeted mechanisms of behavior change, for example, by developing affective interventions.

### The Potential of Affective Interventions for Physical Activity

Interventions that specifically target affect (eg, by using emotion-related behavior change techniques [16]) have the potential to improve the performance of behavior change mHealth apps. Affect is a challenging target for behavior change apps, in part because the relationship between exercise and affect is not straightforward. First, researchers have described a wide variety of separate constructs related to affect [17], including how an individual feels in the moment (incidental affect), their conscious or unconscious assessments of the pleasantness of exercise (affective attitudes and affective associations, respectively), how an individual feels as a result of performing target behavior (affective response), and other aspects of emotional experience [17,18]. Different models of behavior change link these individual constructs differently to health behavior (eg, by listing affect as a component of more general attitudes that factor into behavioral intention [19] or as individual constructs that affect motivation through a variety of conscious and nonconscious processes [17,20-24]). Second, the effect of exercise on individual affective constructs itself varies greatly; for instance, the intensity of exercise has been shown to have an impact on incidental affect during exercise: gentle physical activity is associated with more positive affect, and vigorous exercise is generally associated with a negative affective shift [25].

Despite these complexities, affective constructs are a promising target for intervention. A growing body of work is demonstrating that affective constructs are meaningful predictors of exercise behavior [21]; for instance, positive nonconscious affective evaluations of exercise are associated with higher exercise volume [21] and play a role in both conscious and nonconscious processes regarding exercise behavior [26]. The effect of differences in affective attitudes to exercise can be powerful: 1 study found a 38-minute weekly increase in exercise for every increase of 1 unit on the Feeling Scale (a scale used to measure affective valence [27]) at the 6-month mark of an aerobic exercise program and a 41-minute increase at 12 months [28]. Affective assessments of exercise are even more impactful than other determinants such as self-efficacy [21,29], and 1 study found that sending affectively framed messages yielded more exercise behavior than cognitively framed messages [30].

One affective construct that holds potential for influencing physical activity is *anticipated affective response*, or how an individual expects that a target behavior will make them feel [17]. Generally, affect takes a negative shift during vigorous exercise but rebounds immediately afterward [25]. Much evidence has associated exercise with positive affective states or, at minimum, recovered affective states afterward, starting immediately after exercise and lasting for as long as a day afterward [25,31]. Although the evidence concerning the relationship of positive postexercise affect with exercise intention is somewhat mixed, and there is need for more research on affective responses to exercise to understand individual variation and guard against biased results [32,33], there is some evidence that positive postexercise affect influences affective attitudes toward exercise [34], which themselves are linked to improved exercise behavior [21,34]. Moreover, *anticipated* positive affective response has been positively associated with exercise intention [35] and behavior [17,18], and *priming* people to expect to feel good after exercise has been shown to increase positive affect over the course of exercise [36], postexercise affect [35,36], and exercise intention [35]. The manipulations thus far of anticipated positive postexercise affect have not consistently resulted in increased exercise behavior [35,36], but priming interventions that trigger increased mental elaboration have been associated with more positive postexercise affect [35], suggesting that how a priming intervention is designed may influence its effectiveness. This finding echoes the findings in human-computer interaction literature that reflection at key stages can affect physical activity behavior [37] and suggests that work is needed to understand what forms of interventions can be most effective.

### Text Messaging Physical Activity Interventions

One relatively common form of intervention is SMS text messaging. With >95% of American adults owning a mobile phone capable of sending and receiving SMS text messages [38], SMS text messaging interventions are broadly accessible and can reach more populations than interventions that rely on smartphones [39]. SMS text messages may make up stand-alone interventions or act as components in a larger intervention [39]. Reviews of SMS text messaging interventions for physical activity have encouragingly found positive effects in reducing weight and promoting physical activity [39,40], although evidence is more ambiguous for specific populations [39,41]. Importantly, the efficacy of SMS text messaging interventions rests in part on their content [30] and tailoring, as well as other characteristics [39].

### Study Contribution

In this study, we will conduct a microrandomized trial [42] (trial registration: ClinicalTrials.gov NCT05582369) to evaluate the effect of a stand-alone SMS text messaging intervention designed to manipulate anticipated postexercise affect to increase exercise behavior. Microrandomization is an efficient trial design that enables causal inference with relatively small samples [42,43]. The primary objective is to determine whether manipulating postexercise affect before planned exercise increases calories burned over the 4-hour period when individuals planned to exercise. Our study extends prior research by examining the influence of anticipated postexercise affect not only on exercise intentions but also on actual exercise behavior. The study will contribute empirical findings on the effect of manipulating anticipated postexercise affect on exercise duration and intensity, actual postexercise affect, and other measures of affective attitudes toward exercise, as well as qualitative findings about how such interventions should be designed.

## Methods

We will conduct a 9-week microrandomized trial of an SMS text messaging intervention to make the affective rewards of exercise salient before planned exercise, recruiting adults from the general American population who have an Apple Watch and an iPhone. The field deployment will be followed by interviews to learn about participant experiences with the intervention.

### Intervention Description

The intervention consists of SMS text messages about feeling good *after* exercise, which are sent to participants *before* they exercise to manipulate anticipated postexercise affect. There are 2 types of affective intervention messages: high-reflection messages and low-reflection messages (Table 1). High-reflection prompts ask participants to think about and write down, in the reply message, topics related to feeling good after exercise. Low-reflection SMS text messages do not require a response. They contain messages that participants will write themselves at the time of study enrollment, using fill-in-the-blank templates. The research team will review these messages at the time of enrollment to ensure that they are topical. The fill-in-the-blank templates were developed through a series of pilots on Amazon’s Mechanical Turk, a website that enables paid crowdworkers to complete web-based tasks. The final set consistently generated topical responses from Mechanical Turk respondents. Out of 18 messages (n=9, 50% high-reflection prompts and n=9, 50% low-reflection prompts, enough for participants to complete the study without repeated messages), participants will receive a randomized subset. Having 2 types of SMS text messages will both generate variety for participants and allow us to explore differences in participant experience resulting from different interaction mechanisms; although both are highly personalized, high-reflection messages provoke reflection and mental elaboration in the moment. In other works, mental elaboration or reflection in written form at different planning or pre-exercise stages has been associated with improved postexercise affect [35] and increased step counts [37].

**Table 1:** Primary intervention message types and examples.

Message type	Participant involvement	Example messages
High-reflection messages	Researcher generated; participant response via texting required	What’s your favorite thing about the way exercise makes you feel afterwards? What positive feelings do you want to experience after exercise today? Name three. When was the last time you did some exercise outside? Tell us what it was and one good feeling you felt afterwards.
Low-reflection messages (templates)	Participant generated based on researcher-created fill-in-the-blank templates; no response required	Exercise can be its own reward, because ______ makes you feel _____ afterwards. (Fill in the first blank with something you enjoy about exercise. Fill in the second blank with a good feeling that you get from exercise) Working your body makes you feel _____ after you exercise. (Fill in the blank with a good feeling you get from exercise) Exercise makes you feel _____ afterwards, because ____. (Fill in the first blank with a good feeling that you get from exercise. Fill in the second blank with a reason exercise makes you feel good)

### Study Procedures

#### Microrandomized Trial

**Figure 1:**
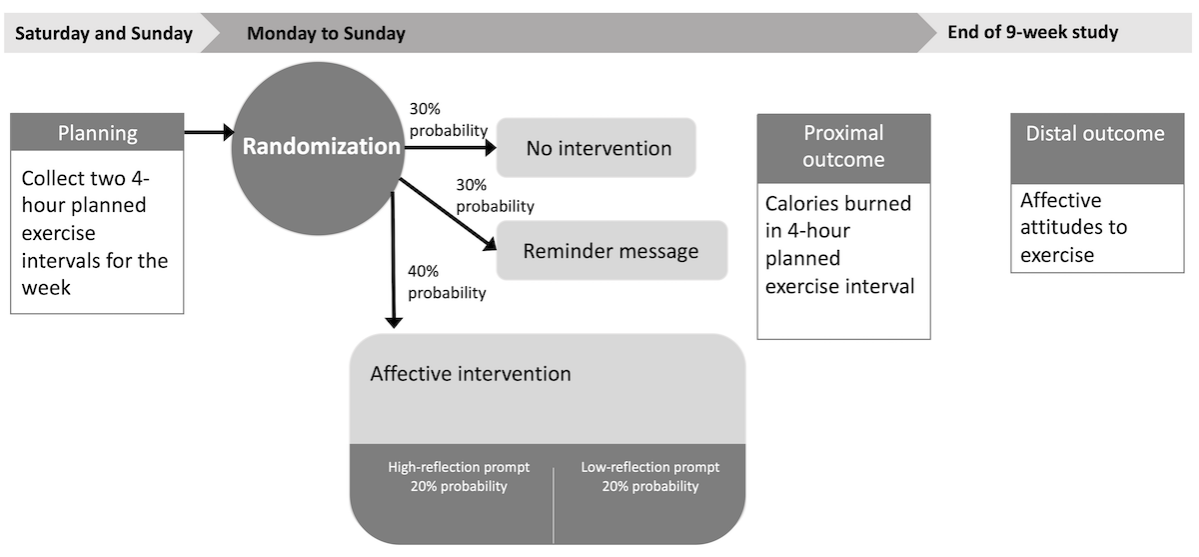
Randomization procedure.

To investigate the effect of the intervention, we will conduct a 9-week microrandomized trial. Participants will complete self-report assessments at enrollment and at the study close. During the 9-week deployment, participants will be randomized twice a week, before planned exercise sessions, to receive an affective intervention message (40% probability), a simple reminder message (30% probability), or no message (30% probability). The study design and measurement points are illustrated in Figure 1 and Table 2. Instruments are listed by study phase in Textbox 1, and specific measures are detailed in Table 3.

**Table 2:** Participant activities and measures by study phase.

Study phase	Participant activities	Measures
Enrollment	Complete low-reflection prompt	Affective Measures SRQ-E^a^ PACES-8^b^
Trial period: 9 wk^c^	Plan 2 exercise sessions/wk Receive and read study SMS text messages Respond to high-reflection prompts via texting Wear Apple Watch Report exercise afterward	Planning Questionnaire Exercise Self-Report Questionnaire
Study close	Closing Questionnaire	Affective Measures SRQ-E PACES-8
Optional interviews	Answer questions about experience with the intervention	N/A^d^

^a^SRQ-E: Exercise Self-Regulation Questionnaire.

^b^PACES-8: Physical Activity Enjoyment Scale.

^c^Affective intervention messages: 40% probability, reminder messages: 30% probability, and no message: 30% probability.

^d^N/A: not applicable.

**Measures used by study phase.**

**Enrollment**

- Consent and enrollment questionnaire (contains affective items)
- Exercise Self-Regulation Questionnaire (SRQ-E)
- Physical Activity Enjoyment Scale (PACES-8)

**Microrandomized trial (9 wk)**

- Planning questionnaire (a version with a question about anticipated postexercise affective state for the next exercise session included will be sent once halfway through study)
- Exercise self-report questionnaire
- Sensor data collection

**Exit**

- Closing questionnaire (contains affective items)
- SRQ-E
- PACES-8

**Table 3:** Specific measures used in the study.

Specific measure or topic; number of times measured during the study		Questionnaires	Items; answer options
**Pre- and postintervention measures**			
	SRQ-E^a^ [44,45]; 2	Consent and enrollment, closing	Refer to the SRQ-E [44]
	PACES-8^b^ [46]; 2	Consent and enrollment, closing	Refer to the PACES-8 [46]
	Baseline activity (self-report); 1	Consent and enrollment	How many days a week do you typically exercise? By exercise, we mean moderate or intense physical activity that lasts for at least 20 minutes (e.g. a brisk 20 minute walk, yoga, running, swimming, etc.); 1 to ≥6
	General affective attitude; 2	Consent and enrollment, closing	How much do you like or dislike exercising?; 7-point scale ranging from I hate exercising to I love exercising
	Affective association; 2	Consent and enrollment, closing	When considering exercise, I feel...; 7-point scale ranging from sad to happy
	Anticipated regret (adapted from the study by Conner et al [47]); 2	Consent and enrollment, closing	If I were not to exercise this week, I would regret it; 7-point scale ranging from very unlikely to very likely
	Anticipated pride (adapted from the study by Conner et al [47]); 2	Consent and enrollment, closing	If I were to exercise this week, I would feel better; 7-point scale ranging from very unlikely to very likely
	General during-exercise affect; 2	Consent and enrollment, closing	While you are doing exercise, how do you typically feel?; free text Rate how good or bad you typically feel while you are doing exercise; Feeling Scale [27]
	General postexercise affect; 2	Consent and enrollment, closing	After you finish exercising, how do you typically feel?; free text Rate how good or bad you typically feel after exercising; Feeling Scale [27]
**Planned exercise information**			
	Planned exercise activity; 9	Planning Questionnaire	What exercise do you plan to do on [day]? Please tell us the activity you plan to do; free text Please tell us roughly how long you plan to do your activity on [day]; free text
	Planned exercise intensity; 9	Planning Questionnaire	Please rate what you expect your overall level of exertion during your planned activity will be. This feeling should reflect how heavy and strenuous you expect the exercise to feel, combining all sensations and feelings of physical stress, effort, and fatigue. Focus on what you expect your total feeling of exertion will be, not any one specific factor?; 10-point scale ranging from 1=*rest* (no exertion at all) to 10=*maximal effort* (I was completely exhausted and it felt almost impossible to keep going)
**Exercise self-report information (after every planned exercise session)**			
	Exercise self-report; 18	Exercise self-report questionnaire	Did you do any exercise today?; Yes, No (conditional) Was the exercise you did today the same as what you originally planned, or different?; Exactly what I had planned; Different from what I had planned; I’m not sure, I can’t remember what I planned (conditional) What exercise did you do today?; free text (conditional) Please let us know why exercise didn’t fit into your day today (conditional) How did the exercise you did differ from what you had planned?; Pushed myself harder; Scaled back my activity; Did the activity in a different place or with different company; Did a different activity; Did the activity at a very different time than I had planned; Other difference
	Exercise exertion; 18	Exercise self-report questionnaire	Thinking back on your physical activity session today, rate your overall level of exertion during it. This feeling should reflect how heavy and strenuous the exercise felt to you, combining all sensations and feelings of physical stress, effort, and fatigue. Focus on your total feeling of exertion, not any one specific factor; 10-point scale ranging from 1=rest (no exertion at all) to 10=maximal effort (I was completely exhausted and it felt almost impossible to keep going)
	Affect during exercise (recollected); 18	Exercise self-report questionnaire	While you were doing your exercise session today, how did you feel overall?; free text Rate how good or bad you felt overall while you were doing your exercise; Feeling Scale [27]
	Postexercise affect (recollected); 18	Exercise self-report questionnaire	After you did your exercise session today, how did you feel overall?; free text Rate how good or bad you felt after your exercise session; Feeling Scale [27]
	Anticipated postexercise affect (next session); 19	Exercise self-report questionnaire and planning questionnaire (with baseline questions)	How do you expect to feel after your next exercise session? Select the number that best represents how good or bad you expect to feel; Feeling Scale [27]
	Unplanned exercise; 18	Exercise self-report questionnaire	Have you done any unplanned exercise since your last planned exercise session (not today’s)?; Yes, No, This was my first planned exercise session for this study (conditional) What was the unplanned exercise you did?; free text

^a^SRQ-E: Exercise Self-Regulation Questionnaire.

^b^PACES-8: Physical Activity Enjoyment Scale.

#### Study Procedures Overview

At enrollment, we will ask participants to write their low-reflection prompts and to self-report demographic and baseline activity–level information. In addition, we will assess participants’ anticipated affect for exercise, their affective association with exercise, and their overall enjoyment of exercise. On top of these measures, we will ask participants to complete the Exercise Self-Regulation Questionnaire [44,45] to measure their intrinsic motivation for exercise and the Physical Activity Enjoyment Scale (PACES-8) [46] to measure their enjoyment of exercise.

For the next 9 weeks, at the start of each week, participants will be asked to list 2 time intervals during which they plan to exercise in the coming week. At the start of each planned exercise time interval, participants will be randomized to receive an affective SMS text message (intervention), a control reminder message, or no message, and at the end of the day, they will be asked to report their actual exercise details (refer to *Exercise Data: Sensor and Self-Report Data* section). Thus, each participant will be randomized to receive an intervention, a control reminder message, or no message 2 times each week.

At the close of the 9-week period, participants will be sent a closing questionnaire that contains questions about their experience with the intervention, all items from the Exercise Self-Regulation Questionnaire and the PACES-8, and the same questions about affect related to exercise that were assessed at baseline.

After the 9-week study period, a subset of participants will be interviewed to gather in-depth data about their experience with the intervention and their perceptions of its impact on their attitudes toward exercise and on exercise behavior. We plan to stratify our interview sample based on participant characteristics such as exercise frequency and participant responses to the closing questionnaire, low-reflection sentences, and responses to high-reflection prompts.

#### Intervention Delivery

Microrandomized trials are designed around *decision points*—times when it may be appropriate and helpful to provide an intervention [42]. As the intervention in this study operates by increasing the salience of postexercise affective rewards, the intervention is likely to be most helpful when participants have an opportunity to exercise but have not yet exercised. To determine these times, participants will complete a planning questionnaire each weekend where they will specify 2 time intervals during which they intend to exercise in the coming week. Participants are asked to give a day and a 4-hour interval (eg, Thursday, 4-8 PM) during which they intend to exercise. We define an intervention decision point as the start of this interval (eg, Thursday, 4 PM). The 4-hour range was selected to be wide enough to give users some flexibility to accommodate small fluctuations in their schedules but narrow enough to still offer a targeted interval to measure outcomes without the addition of too many sources of noise. The planning questionnaire will be sent on Saturdays and again on Sundays and Monday evenings if not yet completed for the week. Participants who do not complete the questionnaire by the end of the day on Monday will not be sent any additional messages for the week.

The planning questionnaire will ask participants both when they intend to exercise and what exercise they plan to do. Participants can do any kind of exercise, as long as their session is at least 20 minutes long and is of moderate or greater intensity. Note that participants may exercise more frequently than twice a week, but only 2 of these sessions—those scheduled through the planning questionnaire—will determine the intervention decision points. Additional, unplanned exercise sessions will be assessed through sensor data and the exercise self-report questionnaire (refer to the *Exercise Data: Sensor and Self-Report Data* section).

The planning questionnaire will be delivered on a weekly, rather than daily, basis to minimize user burden and lower the risk of participant dropout. As the questionnaire requires participants to plan ahead, there is some chance that participants’ plans will change and that they will not exercise at their planned times. If participants change their plans for exercise after sending the planning questionnaire, the intervention may not reach them when they are truly receptive to it. However, the microrandomization of intervention delivery should balance this inaccuracy in delivery across conditions so that the observed effect of the intervention should not be biased in either direction, although it may be attenuated. In addition, the exercise self-report questionnaire, as well as Apple Watch data, will help in detecting whether participants actually exercised during their planned times (refer to *Exercise Data: Sensor and Self-Report Data* section).

#### Reminder Message

The planning questionnaire enables the delivery of the affective intervention messages, but, as a form of implementation intention, it could influence behavior in its own right [48,49]. Specifically, the affective messages may work as reminders of previously made plans, which have been shown to improve the effectiveness of planning [50,51]. We control for these effects by including a reminder message whose delivery will be randomized along with that of the affective intervention messages. The reminder message simply reminds participants to fill out the exercise day questionnaire. As in the case of the affective intervention messages, reminder messages will be sent at the start of the planned exercise interval.

#### Randomization During the 9-Week Trial

##### Randomization Overview

For each planned exercise interval, participants will be randomly assigned to receive an affective intervention message (40% probability), a reminder message (30% probability), or no message (30% probability; Figure 1). If participants are randomized to receive an affective intervention message, they will be further randomly assigned to receive either a high-reflection message or a low-reflection message with equal probability (ie, 20% each). For each participant, as there will be 9 high-reflection messages and 9 low-reflection messages to choose from, the message to be sent will be chosen from the appropriate set randomly without replacement so that participants will not receive the same message twice.

##### Randomization Procedure

As the number of decision points is known in advance (2 sessions/wk, or 18 decision points), we can construct randomized treatment sequences of length 18 for each of the 120 participants. These will be consistent with the 18 decision points per person. Before the start of the project, we created 120 randomization schedules to account for the required sample size, allowing for dropouts. Each schedule consists of a sequence of 18 numbers. To ensure that we achieved the right condition balance across participants, we drew these numbers randomly from a uniform number distribution, then placed them randomly into a hundred 18-number sequences. From the numbers that were randomly selected, we assigned the bottom 30% to the control condition, the next 30% to the reminder condition, and the top 40% to the high- or low-reflection affective message conditions. The result is a randomized sequence of condition assignments for the duration of the study, independent of the time and date of planned exercise sessions (refer to Multimedia Appendix 1 for an example randomization schedule). Across all randomization schedules but not within schedules, the conditions are balanced such that 30% of the planned exercise sessions are assigned to control, 30% to the reminder message condition, and 40% to an affective message condition. We then carried out a second round of randomization on the affective message condition sessions to determine which messages would be sent (eg, high-reflection message 1 and low-reflection message 2). As participants will create a set number of low-reflection messages based on fill-in-the-blank templates, we can randomize message selection before message creation. When a participant enrolls, they will be assigned to a randomization schedule. The actual time and date at which each message is sent will be based on the participants’ responses to the planning questionnaires each week.

#### Text Message Delivery

SMS text messages will be delivered through an SMS text messaging service that enables scheduled sending. Low-reflection prompts, high-reflection prompts, reminder messages, and reminders to wear the Apple Watch and fill out the planning questionnaire will not be personalized with participants’ names or other identifiers (however, for low-reflection prompts, each participant will receive only the messages that they themselves wrote). SMS text messages will be sent from the same mobile phone number each time so that participants recognize it as coming from the study team. The participants’ responses to high-reflection prompts will be received by the same SMS text messaging service and associated with their mobile phone number so that their responses can be tracked and analyzed.

### Measures

Study data will include activity data as measured by Apple Watch (primary outcome) and self-report, questionnaires about affect and exercise, and video chat or telephone interview data about participant experience with the intervention. Questionnaires will be hosted on the Qualtrics platform and sent to participants via SMS text message.

#### Primary Outcome

The primary outcome for the microrandomized trial will be *calories burned*, as assessed by the Apple Watch, over the planned 4-hour exercise periods scheduled through the planning questionnaire. Apple Watch data will be collected through the MyDataHelps platform (CareEvolution, LLC), a secure platform for the collection of sensor (Apple Watch and Fitbit) and self-report data in health research. Participants will install the MyDataHelps app on their mobile phone. As calories burned can be reported by Apple Watch in discontinuous intervals, we will sum the calories burned over the 4-hour planned exercise period. When the intervals of reported calorie data cross the 4-hour planned exercise period boundary, we will assume that the calories burned are equally distributed across the reported interval and include only these calories in the 4-hour planned exercise period. Although Apple Watch’s calories burned measurement has moderate rather than high correlation with other energy expenditure measures [52], calories burned were selected as the primary outcome measure because it enables us to assess participants’ engagement in a greater variety of exercises compared with a more restrictive measure, such as steps. In this study, participants are instructed to perform any moderate to vigorous exercise they choose and to tell us what they plan to do in the planning questionnaire. Other data (described in the next subsection) will provide secondary records of the exercise performed for secondary analyses.

#### Exercise Data: Sensor and Self-Report Data

In addition to exercise being assessed via calories burned, it will also be assessed through several additional Apple Watch metrics, including active minutes, steps, and standing time. These metrics will also be collected through the MyDataHelps mobile app. As with calories burned, we will analyze step data and active minutes in the 4-hour planned exercise period.

On the days that participants plan to exercise, they will be asked to complete an exercise self-report questionnaire in the evening asking whether they exercised that day and, if so, how their activity compared with what they had planned; for example, was it shorter or longer in duration than they had planned? Participants will further be asked about their perceived exertion and affective state during and after exercise and, as a manipulation check, whether they received and read an SMS text message from the study team before exercise. If participants say they did not exercise, they will be asked why.

#### Affect Measures

The exercise self-report questionnaire will ask participants about their affective states during and after exercise, as well as expected postexercise affect for the next exercise session. In addition, at 3 points in the study (baseline, the 3-week check-in, and the end of the study), participants will be asked questions about their affective attitudes toward, during, and after exercise (Table 3).

#### Participant Experience

A closing survey will ask participants about their subjective perceptions of different aspects of the intervention. In addition, interviews will allow us to explore participant experience with the intervention and their perceptions of its impact on their behavior and attitudes.

### Participants

US residents with an iPhone and Apple Watch who are aged at least 18 years and have not been medically advised against physical activity are eligible to participate. Participants will be recruited through web-based channels such as email lists and relevant internet forums. They will be informed about the study and will give consent through a web-based questionnaire.

### Sample Size and Power

Sample size was calculated according to the methods published by Liao et al [43]. A microrandomized trial is an efficient trial design that enables causal inference with relatively small samples [42,43]. For this study, proximal treatment effect was estimated at 0.2, significance set at .05, and power set to 0.9. To power our study for the primary analysis (refer to the next subsection)—a contrast between no message and affective messages—availability was set to 0.7 to exclude reminder messages that are randomized at 30% probability. Given these assumptions, 89 participants are needed. We intend to recruit up to 120, allowing for dropouts.

### Data Analyses

#### Primary Analysis

Our primary interest is the average effect of the affective message intervention across time on calories burned during the planned exercise periods compared with no message being sent. To assess this effect, we will use the centered and weighted least squares method, a standard approach for analyzing microrandomized trial data that allows for robust causal inferences about treatment effects [53] and accounts for correlations in longitudinal data by nesting outcomes within participants. The covariates in our analysis will include time in study, the prior week’s proximal outcome, self-reported baseline activity levels, age, and gender.

#### Secondary and Exploratory Analyses

Additional analyses of the microrandomized trial data will investigate the time trend of the effect of affective messages (ie, the change in the effect of the affective messages over the duration of the study), the contrast between affective messages and reminder messages, and separate over-time and time-trend effects of low-reflection and high-reflection messages. In addition, we will investigate the intervention’s effect on other outcomes, such as step count and active minutes for the 4-hour planned exercise interval, as collected from the participants’ Apple Watches. These analyses, as in the case of the primary analysis, will all be conducted using the centered and weighted least squares method.

In addition to the microrandomized trial analyses, we will conduct a number of exploratory analyses of the affective measure data. We will examine potential changes in the anticipated postexercise affect by investigating changes in pre- and postintervention ratings on the Feeling Scale [27]. Exploratory analyses will include an investigation of changes in other affective measures used and the role of potential moderating factors such as baseline activity level, planned exercise intensity, and exercise intensity on postexercise affective state predictions. In addition to these quantitative analyses, we will thematically analyze interview data in accordance with the principles described in the study by Braun and Clarke [54]. After transcribing the interviews, we will use an iterative inductive approach to explore themes in the data around participants’ underlying needs and the impact the intervention had on their lives. We will triangulate interview responses with observed participant behavior and responses to the closing questionnaire to get a full picture of participant experience with the intervention and insight into appropriate outcome metrics for future iterations.

#### Missingness

Missing data are likely to occur because participants forget to wear their watch or respond to questionnaires. We will send weekly reminders to participants to wear their Apple Watches. For missing wearable and questionnaire data, we will explore whether any observed variables can be used to explain missingness and include these variables in analyses.

### Ethics Approval

This study has been approved by the University of Michigan institutional review board (HUM#00190301).

### Informed Consent and Participation

Informed consent will be obtained through a web-based questionnaire; after filling out an eligibility screener survey, eligible participants will be directed to a separate informed consent questionnaire containing information about the study and contact information for the research team. Although some participant identifiers such as name and mobile phone number must be collected to deliver the intervention and participant incentives, these data will be stored on password-protected servers, and data will be deidentified for analysis. All identifying data will be destroyed by the end of December 2024. Participants will be compensated for their participation with Amazon gift cards. Participants will be given US $10 Amazon gift cards on enrollment and another US $20 Amazon gift card on completion of the study. Participants will additionally be entered into raffles for additional gift cards with every study questionnaire they complete, and they will be given an additional US $10 Amazon gift card if they are interviewed, up to a maximum of US $95 in Amazon gift cards.

## Results

Enrollment began in May 2023, with 57 enrolled by the end of July 2023. The results will be published after data collection is complete.

## Discussion

### Implications and Limitations

Researchers have increasingly highlighted the potential of affective and nonconscious motivators to influence exercise behavior [21,55]. Past work has demonstrated the ability of the manipulations of expected postexercise affect to have an impact on during-exercise affect, postexercise affect, and behavioral intention to exercise [35,36]. Our study investigates the impact of repeating that manipulation in a format that is at once highly personalized and highly scalable and that can be carried forward through time at key decision points for exercise behavior. In addition, we extend prior work by examining the impact of the manipulations of expectations of postexercise affect not only on behavioral intentions but also on actual exercise behavior. The findings will elucidate the influence of this intervention over time on both immediate exercise behavior and overall affective attitudes toward exercise. Given recent findings that affective attitudes toward exercise on a particular day have an impact on exercise behavior over and above affective attitudes toward exercise in general [56], there is high potential for an mHealth intervention manipulating affective attitudes in the moment to influence physical activity. As our study design includes qualitative data, we will also gain insight into participants’ *experience* with the intervention, yielding findings that can shape the design of other systems and uncover unforeseen complications.

Although researchers are increasingly exploring interventions that target affective judgments about physical activity, the emotional consequences of physical activity remain an underused target [57]. Studies that have used SMS text messages that are either ambiguously [58] or explicitly [59] about postbehavior affective states have found affective messaging to be a critical part of effective interventions for such behaviors as physical activity [58] and even water consumption [59]. However, more work is needed to demonstrate the efficacy of interventions in changing both affective attitudes and resultant behavior. Although this study does use some planning- and self-monitoring–related techniques (planning exercise and self-reporting on the consequences, in addition to requiring possession and use of an Apple Watch that also provides feedback on physical activity), our focus is on the manipulation of salience and beliefs about emotional consequences [15]. Our study represents an early test of an intervention targeting these constructs, and our analyses will investigate the impact of messaging that manipulates affective constructs over and above any potential influences of planning and self-monitoring contained in the study.

The findings from this study will shed more light on which directions are profitable to explore within the complex landscape of affective interventions; for instance, if our intervention is effective at changing both attitudes and behavior, further research will be needed to explore the longevity of this effect. By contrast, an observed movement in only 1 of these domains could point toward the need to explore different design techniques to influence anticipated postexercise affect (eg, by investigating whether timing, modality, or accompaniment with other affective interventions helps us to leverage this intervention target to effect behavior change).

Our study has several limitations. A key limitation is that the intervention decision points rely on participants’ understanding on the weekend of when they would be able to exercise in the coming week. Given the messiness of life, participants’ plans or ability to execute these plans may change. Although this dependence on predicted ability to be active is suboptimal, microrandomization should help to ensure that the circumstances that may derail exercise plans will be balanced across the experimental conditions and thus will not bias the estimates of treatment effects. Furthermore, any intervention that is likely to work in the real world will need to be able to deal with a noisy, hard-to-predict environment of people’s day-to-day lives. As such, any potential effects that we see in this study may be conservative estimates of the potential of this type of intervention because future work could substantially improve the timing with which such prompts are delivered.

A second limitation of our study is power with relation to the *form* of affective messaging. Although we have 2 types of affective messages, we will lack power to rigorously assess differences in the effect of each type. However, interviews will help to bridge this gap because the participants’ testimony will give us a rich depiction of their experience with each type of affective message.

Our findings will also be constrained by the limitations of the Apple Watch. Our primary outcome is calories burned; as noted previously, we have selected this measure because the calories burned metric captures a greater variety of exercise types than step count, but our findings will be limited by the ability of the Apple Watch to measure calories burned with precision and accuracy [52]. In addition, not even calories burned can capture every activity. Early Apple Watch models are not waterproof, and even later models that can capture most aquatic activities cannot be used for activities such as diving. These limitations mean that some activities cannot be captured by Apple Watch sensors. However, participants will be able to report them in a questionnaire, enabling us to identify when the sensor data are not reliable.

### Conclusions

Despite the difficulty of promoting physical activity, researchers continue to try new tactics because physical activity is so critical for public health. This study will yield initial evidence about the potential of affective interventions based on anticipated affect to support exercise behavior and will do so based on an intervention that is lightweight, scalable, and can be highly personalized to each individual.

## Appendix

Multimedia Appendix 1 Example randomization schedule.

## Abbreviations

mHealth: mobile health
PACES-8: Physical Activity Enjoyment Scale

## Data Availability

The data sets generated during this study will be available from openICPSR, a service of the Inter-university Consortium for Political and Social Research, after they have been deidentified.
